# Interactys-AI: Toward AI-Driven Structural Mapping of Virus–Host Interfaces for Antiviral Repurposing and Pandemic Preparedness

**DOI:** 10.3390/biom16040541

**Published:** 2026-04-05

**Authors:** Christian Poitras, Ali Harake, Nathalie Grandvaux, Benoit Coulombe

**Affiliations:** 1 Institut de Recherches Cliniques de Montréal (IRCM), Montréal, QC H2W 1R7, Canada; christian.poitras@ircm.qc.ca; 2 Centre de Recherche du Centre Hospitalier de l’Université de Montréal (CRCHUM), Montréal, QC H2X 0A9, Canada; ali.harake@umontreal.ca (A.H.); nathalie.grandvaux@umontreal.ca (N.G.); 3 Department of Biochemistry and Molecular Medicine, Faculty of Medicine, Université de Montréal, Montréal, QC H3C 3J7, Canada

**Keywords:** virus–host interactions, structural AI, AlphaFold 3, protein–protein interactions, drug repurposing, pandemic preparedness

## Abstract

Understanding how viruses engage host cell surfaces is fundamental to infection biology and therapeutic development. While vaccines remain central to prevention, recent global crises have emphasized the need for complementary antiviral strategies that can be mobilized rapidly against both known and emerging pathogens. In this context, artificial intelligence (AI) systems for biomolecular structure prediction, culminating in AlphaFold 3, are reshaping what is experimentally and conceptually achievable. Here, we present “Interactys-AI”, a framework designed to exploit AI-based structural modeling to systematically map virus–host protein–protein interactions (PPIs) and connect them to actionable drug repurposing opportunities. Beyond a technical workflow, Interactys-AI reflects a broader transformation toward predictive and anticipatory antiviral discovery. We describe the conceptual foundations of the platform, its implementation, and its application to influenza A H5N1 hemagglutinin. We further discuss how structural AI may redefine preparedness strategies, highlight current limitations, and outline future directions toward real-time therapeutic hypothesis generation.

## 1. Introduction: A Turning Point in Antiviral Discovery

Virus entry is governed by selective and often highly optimized interactions between viral surface molecules and host receptors or accessory cofactors. Deciphering these molecular interfaces has historically required years of biochemical, structural, and genetic investigation, frequently lagging behind pathogen emergence. In the context of rapidly evolving RNA viruses, this delay can have profound public-health consequences.

The rapid maturation of artificial intelligence (AI)-driven structure prediction is now reshaping this landscape. Since the introduction of AlphaFold-Multimer and subsequent expansions culminating in AlphaFold 3 (AF3), multicomponent biomolecular modeling has reached a level of reliability that permits systematic exploration of candidate protein–protein interfaces at unprecedented scale [1,2,3,4]. Instead of validating one interaction at a time, researchers can computationally prioritize hundreds or even thousands of hypotheses before experimental engagement.

This shift is particularly important for host-targeted antiviral strategies, where actionable knowledge depends on identifying which cellular proteins participate directly in viral engagement and which of those are pharmacologically tractable.

Interactys-AI was conceived within this technological inflection point. Its objective is not merely to perform structural modeling, but to convert predictive structural inference into rapid therapeutic hypotheses. Beyond acceleration, AI alters the epistemology of antiviral research. Instead of relying exclusively on experimentally observed interactions, investigators can now navigate predictive landscapes in which alternative receptor hypotheses, accessory factors, or strain-specific adaptations become computationally testable within days. This inversion, from discovery by observation to discovery by prioritization, has profound implications for how laboratories, funding agencies, and public-health institutions organize response strategies.

This perspective builds conceptually on our previously published large-scale structural screening of H5N1 hemagglutinin–host interactions (our previous work), extending these findings into a generalized translational framework (Interactys-AI) applicable to diverse viral systems.

## 2. Historical Evolution of Virus–Host Interface Mapping

Before the advent of structural AI, the identification of virus–host interfaces relied primarily on experimental approaches (Table 1). Classical virology used receptor-binding assays, mutagenesis studies, crosslinking experiments, and affinity purification followed by mass spectrometry. While these methods remain foundational, they are intrinsically reactive and often pathogen-specific.

Structural elucidation traditionally required years of crystallographic or cryo-EM effort. The discovery of the CD4 receptor for HIV or ACE2 for SARS-CoV required multi-disciplinary convergence over extended timelines. Even when receptor identity was known, resolving interface geometry often lagged behind epidemiological urgency.

High-throughput proteomics later enabled broader interaction screens, including affinity purification–mass spectrometry and proximity labeling strategies. However, these approaches frequently capture indirect associations, depend on specific cellular contexts, and may miss transient or low-affinity extracellular interactions.

The emergence of genome-wide CRISPR screens further accelerated host-factor discovery, revealing functional dependencies rather than direct binding interfaces. Yet functional screens do not provide spatial resolution and may identify regulatory rather than physical interactions.

Structural AI introduces a different epistemic model. Instead of inferring interactions indirectly from phenotypes or network correlations, it predicts physically plausible interfaces directly from sequence information. This transition shifts host-factor discovery from observation-driven to hypothesis-driven and scalable inference.

Interactys-AI is situated within this historical transition: it does not replace experimental approaches but reframes them within a predictive prioritization framework.

## 3. Structural AI Comes of Age for Complex Biology

Over the past several years, AI systems have evolved from predicting monomeric folds to modeling multimeric protein assemblies and heterogeneous complexes involving proteins, nucleic acids, and small molecules with increasing fidelity [1,2]. Community benchmarking initiatives and independent validation studies have demonstrated encouraging concordance between predicted and experimentally resolved interfaces [3,5,6,7,8]. Importantly, these systems produce quantitative confidence metrics such as predicted interface TM-score (ipTM), local interface scoring (LIS), and local interaction area (LIA) that enable systematic ranking rather than qualitative visualization.

For virology, this evolution is transformative. Viral attachment and entry frequently involve not only canonical receptors but also attachment factors, co-receptors, or modulators of membrane dynamics. Many such interactions are transient or context-dependent, making them difficult to capture experimentally. Structural AI provides a uniform inference engine capable of interrogating broad repertoires of host proteins against defined viral antigens under standardized computational conditions.

Another conceptual advance lies in reproducibility. Historically, structural hypotheses emerged from heterogeneous pipelines such as X-ray crystallography, cryo-EM, mutagenesis, each with distinct biases and constraints. AI frameworks, by contrast, apply consistent inference logic across thousands of candidate complexes. This harmonization enables comparative ranking, automated meta-analysis, and integration with orthogonal datasets such as expression atlases or CRISPR screens. Structural modeling thus becomes compatible with high-throughput reasoning, a prerequisite for translational scalability.

Beyond methodological acceleration, structural AI introduces a shift in scientific reasoning. Rather than relying exclusively on experimentally observed interactions, researchers can now explore structured hypothesis spaces in which alternative receptor usage, accessory binding factors, and strain-specific adaptations become computationally testable. This transition has three major implications for how virus–host interactions are discovered, prioritized, and translated into therapeutic hypotheses: (i) prioritization replaces exhaustive discovery, (ii) structural plausibility complements functional screening, and (iii) therapeutic hypothesis generation can occur in parallel with, rather than downstream of, experimental validation.

## 4. Conceptual Framework of Interactys-AI

**Figure 1:**
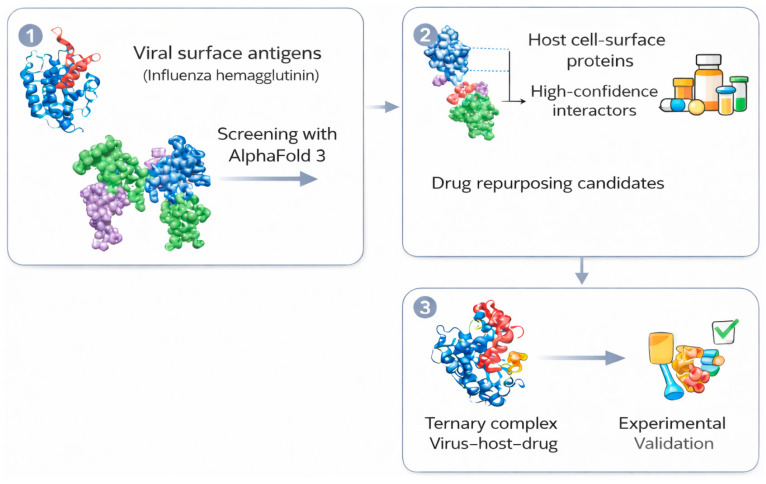
Overview of the Interactys-AI pipeline for AI-driven antiviral discovery. Interactys-AI integrates four successive steps to accelerate host-directed antiviral discovery. (1) Viral surface antigens (e.g., influenza hemagglutinin) are screened against curated human cell-surface proteins using AlphaFold 3 to predict virus–host protein–protein interactions. (2) High-confidence host interactors are prioritized using quantitative interface metrics (ipTM, LIS, LIA) and linked to existing pharmacological knowledge to identify drug repurposing candidates (see Table 2). (3) Candidate drugs are evaluated in silico using AlphaFold 3-based modeling of ternary complexes to assess potential interference with virus–host interactions, and prioritized candidates are forwarded to experimental validation strategies, including interaction assays and functional entry or signaling readouts. This integrated framework enables the rapid identification of repurposable antiviral candidates and strengthens preparedness against emerging viral threats.

Interactys-AI is designed as a translational reasoning system linking four interconnected layers (Figure 1):

(i) Large-scale virus–host structural screening;
(ii) Pharmacological annotation and repurposing mapping;
(iii) AI-based modeling of drug-mediated interaction interference;
(iv) Experimental validation strategy design.

These modules are iterative rather than strictly linear. Experimental outcomes can inform threshold recalibration and ranking logic in subsequent rounds. Over time, such feedback loops may enable adaptive refinement of prioritization criteria, approximating active learning strategies within translational virology.

Together, these layers aim to compress the conceptual distance between molecular recognition and therapeutic hypothesis by directly linking structural prediction outputs to pharmacological and experimental decision-making.

**Table 2:** Interface Metrics and Translational Interpretation.

Metric	Structural Meaning	Translational Relevance
ipTM	Global interface confidence	Initial screening filter
LIS	Local interface residue confidence	Site-specific plausibility
LIA	Buried surface area	Interaction stability proxy
Electrostatic complementarity	Charge compatibility	Drug interference potential
Pocket proximity	Ligand-binding adjacency	Druggability assessment

## 5. Computational Implementation and Screening Strategy

Although Interactys-AI is presented here as a conceptual framework, its feasibility rests on a scalable computational strategy.

At its foundation lies the curated definition of viral antigens and host candidate repertoires. Viral proteins of interest (typically surface glycoproteins or entry mediators) are selected based on biological relevance and epidemiological risk. On the host side, candidate sets can be defined using annotation-driven filtering (e.g., membrane localization, extracellular domains, Gene Ontology classifications), transcriptomic enrichment in target tissues, or integration with prior proteomic interaction datasets.

Each viral antigen is computationally paired against the host candidate repertoire using AF3-based multimer modeling. Predictions are executed in parallelized computational environments, enabling systematic screening. Output models are evaluated using global and local interface confidence metrics. Rather than relying solely on aggregate confidence, Interactys-AI emphasizes interface-specific parameters that better reflect biological plausibility.

Ranking strategies integrate multiple dimensions:

- ipTM or related global confidence indicators;
- Local interface scores;
- Interface surface area;
- Structural complementarity features.

Notably, filtering is not binary. The system generates probability-weighted interactomes in which candidates can be prioritized according to adjustable thresholds. This flexibility is essential when balancing sensitivity versus specificity during outbreak contexts.

Reproducibility derives from uniform computational conditions and transparent threshold logic. Such standardization enables systematic comparison across viral strains, variants, or protein families by ensuring that all candidate interactions are evaluated under consistent computational and scoring conditions.

### 5.1. Quantitative Interface Interpretation and Ranking Logic

A critical determinant of translational value in large-scale structural screening lies not merely in whether a complex can be predicted, but in how its interface is quantitatively interpreted. Global model confidence metrics, while useful, are insufficient proxies for biological relevance. In virus–host contexts, it is the local interaction geometry that determines entry competence, attachment stability, and potential for therapeutic disruption.

Interactys-AI therefore emphasizes multi-parameter interface evaluation. In addition to ipTM-derived global confidence, local interface metrics (such as residue-level confidence, buried surface area, hydrogen-bond density, and electrostatic complementarity) are integrated into composite prioritization schemes. Rather than using fixed cutoffs, dynamic thresholding can be applied depending on the biological question. For example, exploratory mapping may tolerate lower stringency to maximize sensitivity, whereas therapeutic prioritization may require higher interface stability thresholds.

This multi-dimensional scoring approach transforms structural prediction from a binary “interaction/non-interaction” classification into a graded landscape of interaction plausibility, allowing candidates to be ranked and filtered according to quantitative and biologically interpretable criteria. Such landscapes allow ranking, clustering, and comparative analysis across viral variants. Importantly, ranking outputs are transparent and reproducible, permitting threshold recalibration as validation data accumulate.

Moreover, interface interpretation must consider topology constraints. For membrane proteins, predicted interfaces must align with plausible extracellular accessibility. In practice, this requires integrating structural prediction with domain annotation and transmembrane topology data. This additional filtering step substantially reduces false-positive prioritization.

By incorporating these interpretive layers, Interactys-AI shifts structural AI from visualization toward quantitative triage. Key interface metrics and their translational interpretation are summarized in Table 2.

### 5.2. Statistical Calibration and Multiple-Testing Considerations

Large-scale pairwise structural screening inherently raises multiple-hypothesis testing concerns. When hundreds or thousands of viral–host combinations are evaluated, even low false-positive rates can yield substantial numbers of high-confidence yet biologically irrelevant predictions.

Interactys-AI therefore benefits from incorporating empirical calibration strategies. One approach involves benchmarking predicted scores against experimentally validated positive and negative interaction datasets to derive empirical confidence thresholds. Another involves permutation testing, where viral sequences are paired with randomized host controls to establish background score distributions.

At the current stage, such calibration strategies have not yet been systematically applied to the H5N1 dataset presented in our previous study [9], but represent a critical next step toward quantitative interpretability. Preliminary analyses suggest that empirical benchmarking against known interaction datasets will be essential to refine threshold selection and reduce false-positive prioritization in large-scale screening contexts.

## 6. AI-Based Virus–Host Interaction Mapping

The primary output of structural screening is a ranked virus–host interactome. Unlike experimentally derived interaction maps, which often reflect specific cell types or assay biases, AI-derived landscapes represent hypothesis spaces that can be interrogated under consistent structural assumptions.

This triage capacity reshapes experimental planning by enabling laboratories to focus validation efforts on a prioritized subset of structurally plausible interactions, thereby reducing time and resource constraints. During emerging outbreaks, resources, biosafety constraints, and primary material availability are limiting. Computational prioritization enables laboratories to concentrate efforts on the most structurally credible interaction scenarios.

Recent large-scale applications of AF-derived metrics demonstrate improved discrimination between biologically plausible and spurious complexes [5,6,10]. By converting structure prediction into ranked inference, Interactys-AI transforms modeling into decision-support infrastructure.

### 6.1. Structural Determinants of Viral Engagement Beyond Canonical Receptors

Viral attachment is often presented as a single receptor–ligand interaction; however, increasing evidence indicates that entry frequently involves multi-component surface microenvironments. Co-receptors, glycan scaffolds, adhesion molecules, and signaling adaptors can collectively modulate viral binding avidity and internalization efficiency.

Structural AI enables systematic exploration of such accessory axes. By screening viral glycoproteins against broad extracellular repertoires, one may identify candidate stabilizers of attachment complexes or modulators of membrane clustering. These interactions may not independently mediate entry but could enhance local viral concentration or alter membrane curvature, indirectly influencing infectivity.

Importantly, structural modeling allows discrimination between direct steric interfaces and distant regulatory associations inferred from systems biology datasets. In doing so, it refines network-level observations into spatially explicit hypotheses.

This spatialization of interactomes represents a conceptual advance by converting abstract interaction networks into physically constrained models that can be directly tested through mutagenesis or targeted inhibition strategies: it converts abstract interaction maps into physically constrained models that can guide mutagenesis or targeted inhibition strategies.

### 6.2. Illustrative End-to-End Workflow Example

To concretize the Interactys-AI framework, we provide here an illustrative example reflecting a typical analytical trajectory.

Layer 1: Structural Screening:

The hemagglutinin (HA) glycoprotein of influenza A H5N1 is screened against a curated panel of human cell-surface proteins. Among predicted complexes, several candidates exhibit elevated interface confidence scores (e.g., ipTM > 0.6) combined with favorable local interface metrics (LIS, LIA), suggesting structurally plausible engagement.

Layer 2: Pharmacological Annotation:

High-confidence host interactors are cross-referenced against curated pharmacological databases (e.g., DrugBank, Open Targets, ChEMBL, DGIdb). This step identifies proteins with known ligands, inhibitors, or clinically approved drugs, thereby establishing immediate translational relevance.

Layer 3: Structural Interference Modeling:

Selected host–virus complexes are re-modeled in the presence of candidate ligands. Comparative structural analysis between ligand-free and ligand-bound states enables identification of potential steric occlusion, altered interface geometry, or reduced complementarity.

Layer 4: Experimental Prioritization:

Top-ranked hypotheses are forwarded to validation pipelines, including binding assays (e.g., TR-FRET, surface plasmon resonance) and functional entry assays (e.g., pseudovirus systems). Structural ranking thus directly informs experimental resource allocation.

This example illustrates how Interactys-AI compresses the path from structural prediction to experimentally testable antiviral hypotheses.

## 7. H5N1 Illustration: Structural Insight into a High-Risk Virus

This perspective builds conceptually on our previously published dataset of H5N1 HA–host interactions [9], extending it into a generalized translational framework (Interactys-AI). Influenza A H5N1 provides a compelling test case. Hemagglutinin (HA) mediates viral attachment through sialic acid recognition, yet receptor usage is modulated by strain-specific adaptations and host context. While canonical α2,3-linked sialic acids dominate avian tropism, zoonotic transmission events raise concerns about adaptive shifts toward human-compatible receptor environments.

Structural AI enables exploration beyond canonical glycan recognition by systematically evaluating potential protein–protein interactions that may act as accessory attachment or stabilization factors. By screening HA against curated human surface proteins, candidate accessory interactions or modulatory host axes can be computationally prioritized. Such hypotheses extend beyond classical receptor biology and may reveal host components influencing viral attachment stability, membrane clustering, or signaling crosstalk.

Host-directed targeting offers conceptual advantages. Whereas viral proteins mutate rapidly, host proteins are genetically stable. Identifying structurally plausible host engagement points therefore creates opportunities for therapeutic strategies less susceptible to viral escape.

In this illustrative context, structural inference identifies candidate host pathways with known pharmacological modulators, highlighting how predictive modeling can intersect directly with translational feasibility. These observations are consistent with previous reports describing accessory host factors involved in viral entry processes.

### 7.1. Representative High-Confidence Host Interactors

A subset of top-ranked candidate interactors is presented in Table 3 to illustrate the structure of the output and prioritization logic.

Representative top-ranked candidates include IGHV2-5, BMP2, PROCR, ICAM1, and LY6G6D, illustrating the diversity of functional classes captured by the structural screening approach.

First, predicted interactors extend beyond classical viral receptors and include proteins involved in signaling, adhesion, and immune modulation. This supports the hypothesis that viral attachment may involve multi-component surface environments rather than single receptor interactions [11,12,13].

Second, several candidates are already pharmacologically targeted in other disease contexts, such as signaling pathways associated with predicted interactors such as BMP2, creating immediate opportunities for drug repurposing.

### 7.2. Biological Interpretation

The diversity of predicted host interactors suggests that H5N1 HA may engage a distributed interaction landscape involving: (i) membrane receptors influencing cell signaling; (ii) adhesion molecules contributing to viral stabilization; and (iii) immune-related proteins modulating host recognition.

Such interactions may not independently mediate viral entry but could enhance binding avidity, promote membrane clustering, or facilitate downstream internalization processes.

This perspective aligns with emerging views of viral entry as a systems-level process, in which multiple weak or transient interactions collectively shape infectivity.

### 7.3. Translational Implications

From a therapeutic standpoint, host-directed targeting offers strategic advantages by focusing on genetically stable cellular proteins, thereby reducing the likelihood of rapid viral escape through mutation. Unlike viral proteins, host factors are genetically stable, reducing the likelihood of rapid resistance through viral mutation.

Moreover, structural prioritization enables identification of druggable nodes within the host interaction network, thereby bridging molecular prediction and clinical feasibility.

In this context, Interactys-AI does not merely identify interactions but organizes them into actionable therapeutic hypotheses, prioritizing targets that combine structural plausibility with pharmacological accessibility. Importantly, this prioritization strategy bridges predictive structural biology with actionable therapeutic hypotheses, addressing a critical bottleneck in antiviral discovery.

## 8. Linking Structural Predictions to Drug Repurposing

A distinguishing feature of Interactys-AI is the immediate integration of structural predictions with pharmacological knowledge.

Following structural prioritization, host interactors are systematically mapped to established drug-target databases, including DrugBank, Open Targets, ChEMBL, and DGIdb [14,15,16,17,18,19,20,21,22,23]. These resources provide information on known ligands, clinical-stage compounds, and approved drugs.

For example, predicted interactors such as EGFR or CD47 are already targeted by clinically approved or investigational agents in oncology. Their structural proximity to viral binding interfaces raises the possibility that ligand binding could interfere with virus–host engagement, either directly (steric occlusion) or indirectly (allosteric modulation).

Importantly, this approach shifts drug repurposing from pathway-level inference toward structure-informed prioritization by evaluating whether druggable proteins are physically positioned to interfere with virus–host interfaces. Instead of asking whether a protein is involved in a relevant pathway, Interactys-AI evaluates whether a druggable protein is physically positioned to disrupt viral attachment.

### Example of Structure-Informed Repurposing Logic

A candidate host protein predicted to interact with HA is first evaluated for druggability. If the protein contains a known ligand-binding pocket proximal to the predicted viral interface, candidate compounds can be prioritized for structural interference modeling.

Subsequent modeling of ligand-bound complexes may reveal: (i) direct overlap between the drug-binding site and the viral interface; (ii) conformational shifts reducing interface complementarity; and (iii) steric hindrance of viral contact residues.

Such structure-informed reasoning provides a mechanistic basis for repurposing decisions, strengthening translational confidence.

## 9. Virtual Assessment of Interaction Disruption

A further conceptual layer involves modeling ternary assemblies—virus, host, and candidate drug—to assess potential interference with viral engagement.

Although quantitative free-energy prediction remains imperfect, comparative structural modeling between drug-free and drug-bound states can reveal informative trends. These include:

- Steric occlusion of viral interface residues;
- Reduced interface complementarity;
- Altered local confidence metrics;
- Displacement of key interaction determinants.

Even qualitative shifts in predicted geometry may justify experimental prioritization. As training datasets expand to include more ligand-bound complexes, predictive fidelity is expected to improve [24,25,26].

Future integration with molecular dynamics simulations or ensemble-based structural sampling may further refine this layer, bridging static inference and dynamic biological reality.

## 10. Scalability and Variant Propagation

One of the most transformative implications of AI scalability lies in preparedness.

Structural libraries of pre-computed virus–host interaction landscapes could be generated for viral families with known zoonotic potential [27,28,29,30,31]. When new variants emerge, mutational updates could be computationally propagated across existing structural frameworks. Interface re-scoring would allow rapid reassessment of previously prioritized therapeutic hypotheses.

Such infrastructure would resemble genomic surveillance databases in that continuously updated structural models could be queried, compared across variants, and integrated into public-health decision frameworks. Mutation-aware re-ranking pipelines could dramatically shorten the interval between variant detection and therapeutic triage.

In this vision, AI modeling becomes embedded within public-health infrastructure rather than operating solely as retrospective research.

### Mutation-Aware Interface Recalibration

RNA viruses exhibit high mutational plasticity. Structural AI provides a platform for mutation-aware interface recalibration. When new viral variants emerge, sequence substitutions can be rapidly projected onto existing structural models. Comparative interface analysis allows quantification of predicted changes in interaction strength, surface complementarity, or residue-level confidence.

Such differential scoring may indicate whether previously prioritized host axes remain viable therapeutic targets. Conversely, it may reveal variant-specific vulnerabilities that were not apparent in ancestral strains.

Importantly, mutation propagation analysis requires careful interpretation. Single-residue substitutions may alter local geometry without fully disrupting binding. Therefore, variant assessment should combine structural scoring with evolutionary conservation analysis and, where possible, experimental validation.

Embedding mutation-aware recalibration into preparedness pipelines transforms structural AI into a dynamic rather than static resource.

## 11. Toward a Structural Early-Warning System

Genomic surveillance has become central to pandemic preparedness. However, genomic variation alone does not immediately reveal functional consequences. Structural AI offers the possibility of establishing a complementary structural surveillance layer [27,28,29,30,31].

In such a system, viral sequences deposited into public databases could automatically trigger:

i. Structural modeling of key surface antigens;
ii. Propagation of mutations onto precomputed host-interaction landscapes;
iii. Automated re-ranking of candidate host interfaces;
iv. Cross-referencing with pharmacological databases.

Within days of variant detection, updated structural interactome maps could be generated, enabling rapid reassessment of host interaction hypotheses and therapeutic prioritization. This would enable early identification of potential shifts in receptor usage, altered binding geometry, or emerging therapeutic vulnerabilities.

Such a structural early-warning system would not predict epidemiological spread but could inform therapeutic prioritization, experimental design, and public-health resource allocation.

Importantly, this vision requires:

- Scalable compute infrastructure;
- Standardized confidence reporting;
- Shared open datasets;
- Interdisciplinary governance.

If implemented collaboratively, structural AI could become embedded within international preparedness networks alongside genomic and epidemiological monitoring.

## 12. Integration with Systems Virology

Structural inference gains power when integrated with orthogonal datasets. Proteomic interaction maps, CRISPR screens, transcriptomic atlases, and network modeling initiatives have produced extensive candidate repertoires [32,33,34,35,36,37]. Structural AI can provide spatial interpretation of these datasets.

Viruses often exploit multiprotein assemblies rather than isolated factors. Structural context helps discriminate direct physical interfaces from indirect regulatory associations. By bridging abstract network topology with three-dimensional geometry, Interactys-AI contributes mechanistic clarity to systems-level observations.

## 13. Limitations and Responsible Use

Despite its promise, structural AI has limitations.

Predictions may not fully capture glycosylation states, membrane curvature, lipid microdomains, or competitive binding dynamics. Stoichiometry ambiguity and conformational plasticity (particularly for viral glycoproteins) introduce additional uncertainty. Training data biases may influence confidence calibration.

Large-scale screening inevitably produces false positives. Experimental validation remains indispensable. AI should guide experimental prioritization, not replace empirical investigation.

Operationally, scalable prediction may create unrealistic expectations of immediate therapeutic solutions. Transparent reporting of confidence metrics, threshold criteria, and limitations is essential to maintain scientific and public trust.

Responsible deployment requires balance between innovation and restraint.

### Calibration, Bias, and Interpretive Constraints

While AF3 and related systems have achieved remarkable performance, their training datasets are enriched for certain structural classes and underrepresent others, including membrane-embedded assemblies and heavily glycosylated complexes. Viral glycoproteins often rely on glycan shielding and conformational flexibility not fully captured by static predictions.

Confidence metrics must therefore be interpreted probabilistically rather than deterministically. High interface confidence does not guarantee biological relevance, and low confidence does not necessarily imply absence of interaction.

Additionally, large-scale screening raises multiple-testing considerations. When thousands of candidate pairs are evaluated, statistical calibration becomes essential to avoid inflated prioritization.

Future development may involve benchmarking predicted interactomes against experimentally derived datasets to derive empirical calibration curves. Such benchmarking would improve threshold selection and reduce systematic bias.

Recognizing these methodological constraints is central to responsible implementation.

## 14. Perspective: What Is Next?

We anticipate several near-term developments:

(i) Structural AI-derived hypotheses will become routine components of outbreak response.
(ii) Drug repurposing logic will increasingly originate from interface geometry rather than solely from pathway membership.
(iii) Collaborative computational ecosystems will dominate antiviral innovation.

Workforce evolution will be equally important. Biologists, clinicians, and computational scientists must develop shared interpretive frameworks enabling rapid translation of predictions into actionable experiments.

### 14.1. Structural AI as Translational Infrastructure

The broader implication of Interactys-AI lies not only in methodological innovation but in infrastructural transformation. Pandemic preparedness has traditionally relied on genomic surveillance and epidemiological modeling. Structural AI introduces a third pillar: predictive molecular cartography.

If deployed collaboratively and transparently, such platforms could maintain continuously updated structural landscapes of viral families with zoonotic potential. These resources could be integrated with public-health agencies, pharmaceutical consortia, and academic networks to accelerate coordinated response.

However, infrastructure requires governance. Standardized reporting of confidence metrics, open access to ranking logic, and clear delineation between hypothesis and validation are essential to prevent overinterpretation.

Embedding structural AI within public-health ecosystems therefore demands not only computational power but shared scientific norms.

### 14.2. Ethical and Communication Considerations

Structural AI carries reputational risk if overinterpreted. During crises, predictive outputs may be misrepresented as confirmed biological mechanisms. Care must therefore be taken in public communication to distinguish hypothesis from validation.

Moreover, large-scale screening may identify host proteins with essential physiological roles. Therapeutic targeting strategies must weigh antiviral benefit against systemic risk.

Open reporting of uncertainty, confidence ranges, and validation status should accompany any translational recommendation derived from AI prediction.

Responsible integration of structural AI requires alignment between computational ambition and biomedical caution.

## 15. Conclusions

Interactys-AI is more than a workflow; it represents a conceptual reorientation in antiviral research, shifting from reactive experimentation to anticipatory, structure-guided prioritization.

Structural AI has matured to a point where systematic virus–host interface exploration is computationally feasible at large scale. When integrated with pharmacological annotation, mutation-aware recalibration, and systems-level datasets, this approach creates a translational bridge between molecular geometry and therapeutic hypothesis generation.

Yet technological capability must be paired with methodological rigor. Calibration, validation, and transparent communication are prerequisites for responsible deployment.

The future of antiviral discovery may increasingly rely on continuously updated structural landscapes that evolve in parallel with viral genomes. If developed collaboratively and embedded within preparedness infrastructure, structural AI could shorten the time between variant emergence and therapeutic triage from months to days.

The challenge ahead is not merely technical, it is organizational and epistemological. Converting predictive molecular cartography into coordinated public-health action will require shared standards, interdisciplinary fluency, and sustained institutional commitment.

In this context, Interactys-AI illustrates how predictive structural biology can evolve from a modeling discipline into a core component of translational and preparedness infrastructure.

## Figures and Tables

**Table 1:** Comparative Approaches to Virus–Host Interaction Discovery.

Approach	Resolution	Throughput	Direct Physical Interface?	Speed During Outbreak	Limitation
Affinity purification–MS	Moderate	Medium	Often indirect	Moderate	Context-dependent
CRISPR screening	Functional	High	No	Moderate	No spatial detail
Cryo-EM/X-ray	Atomic	Low	Yes	Slow	Labor intensive
Structural AI screening	Atomic-scale prediction	Very high	Yes (predictive)	Rapid	Requires validation

**Table 3:** Representative top-ranked human cell surface proteins predicted to interact with H5N1 hemagglutinin.

Protein Name	Gene Symbol	ipTM	LIS	LIA	Functional Annotation	Druggability
Immunoglobulin heavy variable region	IGHV2-5	0.71	High	High	Immune recognition	Indirect targeting possible
Bone morphogenetic protein 2	BMP2	0.60	High	High	TGF-β signaling ligand	Targetable (pathway modulation)
Endothelial protein C receptor	PROCR	0.60	Moderate–High	High	Coagulation, endothelial signaling	Targeted in anticoagulation pathways
Intercellular adhesion molecule 1	ICAM1	0.54	High	High	Adhesion, viral entry facilitation	Targetable (anti-inflammatory strategies)
LY6/PLAUR domain-containing protein 6D	LY6G6D	0.40	High	Moderate	Immune modulation (less characterized)	Potential exploratory target

Values correspond to representative high-ranking interactions derived from large-scale structural screening of ~1000 human cell surface proteins using AlphaFold-based modeling [9]. The complete dataset is available in the original study.

## Data Availability

The data presented in this study are available within the article. Further inquiries can be directed to the corresponding author.

## Abbreviations

AF3	AlphaFold 3
AI	Artificial Intelligence
GO	Gene Ontology
HA	Hemagglutinin
LIS	Local Interface Score
LIA	Local Interaction Area
PPI	Protein–Protein Interaction
